# The Role of Key Glycolytic Enzymes in the Diagnosis, Treatment, and Immune Microenvironment of Colorectal Cancer

**DOI:** 10.1155/humu/9989417

**Published:** 2025-11-19

**Authors:** Haijuan Gu, Chunhua Liu, Erdong Cai, Yongfeng Cao, Jibin Liu

**Affiliations:** ^1^Department of Pharmacy, Affiliated Tumor Hospital of Nantong University & Nantong Tumor Hospital, Nantong, Jiangsu, China; ^2^Department of Gynecology Oncology, Affiliated Tumor Hospital of Nantong University & Nantong Tumor Hospital, Nantong, Jiangsu, China; ^3^Cancer Research Center Nantong, Affiliated Tumor Hospital of Nantong University & Nantong Tumor Hospital, Nantong, Jiangsu, China; ^4^Department of Internal Medicine, Affiliated Tumor Hospital of Nantong University & Nantong Tumor Hospital, Nantong, Jiangsu, China; ^5^Institute of oncology, Affiliated Tumor Hospital of Nantong University & Nantong Tumor Hospital, Nantong, Jiangsu, China

**Keywords:** carbohydrate metabolism, colorectal cancer, glycolysis, glycolysis-related enzymes, immune microenvironment

## Abstract

Colorectal cancer is acknowledged as the fifth most common cause of cancer-related mortality, presenting significant challenges for patient outcomes due to its relatively gradual progression and the subtle nature of its initial symptoms. Carbohydrates, essential nutrients in cellular function, participate in various metabolic processes, including glycolysis, oxidative phosphorylation, and the pentose phosphate pathway. Recent studies have established that irregularities in carbohydrate metabolism play a critical role in tumor cell growth, development, and treatment resistance. Glycolysis serves as a crucial regulatory component of metabolism in cancer cells, influencing cell growth, proliferation, and functionality by modifying carbohydrate utilization. By diminishing oxidative phosphorylation activity and enhancing energy production through glycolysis, tumor cells augment their proliferative capacity and partially evade immune responses. As a result, glycolysis significantly contributes to tumor progression. We have comprehensively outlined the functions of glycolysis and its key enzymes concerning the diagnosis, treatment strategies, and immune microenvironment of colorectal cancer, with the goal of delivering innovative insights and perspectives for the clinical management and diagnosis of this condition.

## 1. Introduction

Colorectal cancer (CRC) represents a widely occurring malignant tumor of the digestive system globally and ranks as the fifth primary cause of cancer-related fatalities, with its incidence consistently on the rise [1–3]. Because the progression of colorectal cancer is relatively slow and the early symptoms are often subtle, numerous patients tend to receive a diagnosis only after the disease has progressed to an advanced stage, creating considerable challenges for early detection and screening initiatives [4]. While individuals with early-stage CRC can attain positive prognoses through a combination of treatment strategies, including surgery and chemoradiotherapy, nearly 25% are found to have advanced metastases, which significantly reduces the 5-year survival rate for those with metastatic CRC to about 14% [5]. As a result, enhancements in treatment effectiveness and survival rates for advanced CRC patients remain inadequate. This pressing issue highlights the necessity for early CRC screening and signals an urgent requirement for thorough investigation into the molecular mechanisms that drive CRC development and advancement, aiming to identify new therapeutic targets and intervention strategies capable of improving patient outcomes and survival rates.

**Figure 1:**
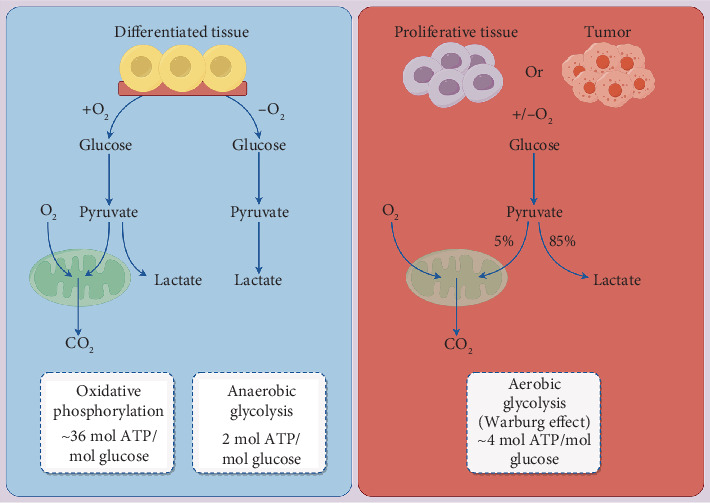
Tumor cells generate a large amount of energy through the Warburg effect.

A considerable amount of research suggests that cancer cells persistently modify their glucose metabolism in settings marked by vascular issues, severe nutrient deficiencies, and low oxygen levels to ensure their survival. This metabolic adaptation is influenced by a variety of stresses stemming from both internal dynamics and external environmental factors throughout tumor development, progression, metastasis, and the onset of resistance to treatment [6–8]. Carbohydrates, recognized as one of the three core nutrients within cells, engage in metabolic processes that encompass glycolysis, oxidative phosphorylation, and the pentose phosphate pathway, all of which together generate vital energy necessary for life functions [9]. In the absence of oxygen, normal cells convert glucose into lactate in a process termed glycolysis, which produces a limited amount of ATP [10–12]. Glycolysis can be divided into two phases: initially, glucose is degraded into pyruvate, and then pyruvate is further transformed into lactate. When oxygen is available, the pyruvate formed from glycolysis enters the mitochondria, undergoes the tricarboxylic acid cycle, and progresses to oxidative phosphorylation, thus yielding a significant quantity of energy to satisfy cellular requirements. This pathway serves as the primary mechanism whereby human cells generate energy [13]. Importantly, the rate of glycolysis in cancer cells is markedly elevated; they primarily favor the glycolytic route for energy production, not only in low oxygen conditions but also in the presence of oxygen. This phenomenon, termed the Warburg effect, reflects a defining trait of many cancer cells, which preferentially rely on aerobic glycolysis for their energy needs [14, 15]. In the glycolytic pathway, glucose metabolization yields less ATP in comparison to oxidative phosphorylation [16]. Nevertheless, tumor cells exhibit a strong preference for glycolysis. This metabolic transition from oxidative phosphorylation to glycolysis is a defining trait of cancer cells and illustrates a notable phenomenon of metabolic reprogramming within these cells [17]. Although the glycolytic pathway is less efficient in energy generation compared to mitochondrial oxidative phosphorylation, malignant tumor cells benefit from a robust glycolytic metabolism. The rate of ATP production through glycolysis is nearly 100 times greater than that achieved via oxidative phosphorylation [18]. Consequently, tumor cells utilize substantial amounts of glucose through glycolysis to swiftly produce adequate ATP for their energy needs and to acquire intermediate metabolites essential for the synthesis of crucial building blocks necessary for their growth and proliferation, thus maintaining redox balance to fulfill their heightened anabolic requirements [19]. These findings reveal the metabolic characteristics of tumor cells, which can generate substantial energy through the Warburg effect to promote tumor progression (Figure 1).

## 2. Glycolysis and the Biochemical Characteristics of Its Key Enzymes

### 2.1. Glycolysis in Non-Tumor Cells and Tumor Cells

**Figure 2:**
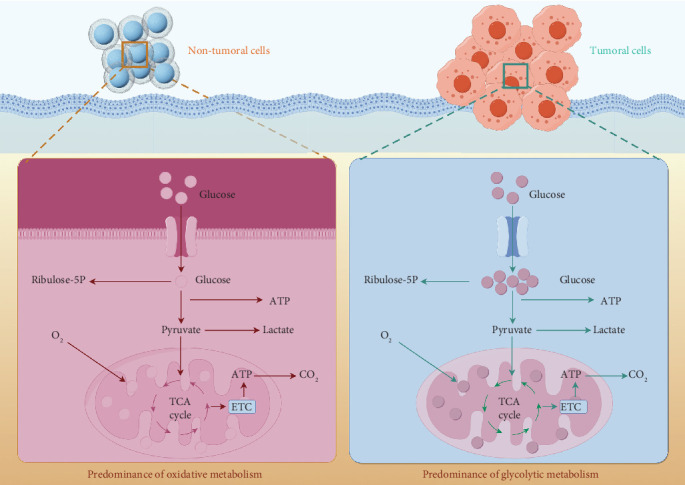
Differences in glucose metabolism between tumor cells and non-tumor cells.

Regarding glucose metabolism, there are notable distinctions between non-tumor cells and tumor cells [20]. Non-tumor cells mainly depend on oxidative metabolism for generating energy. When glucose enters these cells, it is primarily transformed into pyruvate, which subsequently moves into the mitochondria under aerobic conditions to participate in the tricarboxylic acid (TCA) cycle and the electron transport chain (ETC), ultimately resulting in the generation of significant quantities of ATP and carbon dioxide. Only a small amount of pyruvate converts to lactate, while some glucose is processed via the pentose phosphate pathway to produce ribulose-5-phosphate (R5P), which supplies precursors for nucleotide formation and other cellular components. In contrast, tumor cells demonstrate unique metabolic features. Even when oxygen is plentiful, glucose entering tumor cells largely undergoes glycolysis, with most pyruvate converting to lactate, while only a small fraction enters the mitochondria for the TCA cycle and oxidative phosphorylation. This process is known as “aerobic glycolysis” or the Warburg effect. Although this metabolic route is less efficient in generating ATP, it permits quick energy production and the formation of reactive intermediates, while also yielding considerable amounts of ribulose-5-phosphate through the pentose phosphate pathway, thus facilitating the enhanced proliferation and biosynthetic functions of tumor cells. As a result, the essential differences in glucose metabolic pathways between tumor and non-tumor cells create a distinctive metabolic environment that fosters the growth and proliferation of tumor cells (Figure 2).

### 2.2. The Role of Key Enzymes in Glycolysis

The principal enzymes involved in glycolysis are crucial for cellular metabolism. Pyruvate dehydrogenase kinases (PDKs) oversee the transformation of pyruvate into acetyl-CoA through the mitochondrial pyruvate dehydrogenase complex (PDHC). Actively functioning as the rate-limiting enzyme within PDHC, PDKs provide a link between glycolysis and the tricarboxylic acid cycle. In the context of various diseases, PDKs significantly impact cellular metabolism, especially concerning mitochondrial operations. They play a vital role in managing metabolic reprogramming, mitochondrial activity, and cellular functions in both tumor-related and non-tumor-related conditions [21]. Heat shock protein 90 (Hsp90) is integral to tumor development and advancement. Research in gastric cancer has demonstrated its interaction with enzymes associated with glycolysis, resulting in the formation of multi-enzyme complexes that increase glycolytic efficiency and manage the spatial arrangement of glycolytic enzymes at the cell's periphery and lamellipodia, which in turn fosters cell migration and maintains stemness [22]. In the plant kingdom, heterodimers comprised of cyclins (Cycs) and cyclin-dependent kinases (CDKs) regulate two critical enzymes of glycolysis, phosphofructokinase (PFK) and pyruvate kinase (PK), thereby affecting the glycolytic pathway to uphold cellular energy balance [23]. In addition to the aforementioned enzymes, many glycolytic enzymes exhibit non-canonical functions. For instance, certain glycolytic enzymes are implicated in processes such as transcriptional regulation, autophagy, and epigenetic modifications. Although the mechanisms underlying their non-canonical activities remain incompletely understood, these findings offer novel insights into the relationship between cellular metabolism and various physiological and pathological processes [24]. Furthermore, metabolic enzymes including hexokinase, pyruvate kinase, and lactate dehydrogenase play a significant role in the glycolytic reprogramming associated with tumors. These enzymes are essential not only for the development of tumors and the sustenance of tumor cell viability but also in influencing the tumor microenvironment by affecting immune evasion, angiogenesis, and other mechanisms that alter the tumor environment [25].

### 2.3. The Relationship Between Glycolysis and Colorectal Cancer

An increasing body of evidence suggests that glycolysis is closely linked to the development and progression of colorectal cancer. The long non-coding RNA (lncRNA) FTX is significantly upregulated in the cancer tissues, serum, and colorectal cancer cell lines of patients with colorectal cancer. It can sponge miR-215-3p, upregulate YAP1, and thereby promote aerobic glycolysis, cell proliferation, migration, and invasion, playing a crucial role in the progression of colorectal cancer [26]. Research has found that the abnormal expression of various non-coding RNAs (ncRNAs), such as miRNAs, lncRNAs, and circular RNAs (circRNAs), is closely related to glycolysis in colorectal cancer. These ncRNAs influence the glycolysis process through different regulatory pathways, thereby affecting tumor cell proliferation, invasion, chemotherapy resistance, and immune evasion [12]. In terms of metabolism, HOTAIR can regulate the expression of enzymes related to glycolysis and glutaminolysis in colorectal cancer cells, such as PFKFB4, PGK1, and LDHA, directly impacting the production of lactate and glutamate. This indicates its significant role in maintaining the bioenergy and biomass of tumor cells [27]. LGALS4 is significantly downregulated in colorectal cancer, and its overexpression can inhibit cell growth, induce cell cycle arrest, enhance 5-fluorouracil-induced apoptosis, and suppress aerobic glycolysis, suggesting its potential as a novel therapeutic target for colorectal cancer [28]. Additionally, MYG1 is upregulated during the progression of colorectal cancer, promoting glycolysis and tumor development through nuclear-mitochondrial collaboration. It recruits the HSP90/GSK3*β* complex to promote PKM2 phosphorylation, activating MYC-mediated glycolysis, while simultaneously suppressing mitochondrial respiration and apoptosis [29].

## 3. Pathological Mechanisms of Glycolytic Key Enzymes in Colorectal Cancer

### 3.1. The Role of Glycolytic Key Enzymes in the Metabolism of Colorectal Cancer Cells

The crucial enzymes involved in glycolysis significantly contribute to the metabolic processes of colorectal cancer cells. In the context of colorectal cancer, the expression of SIRT1, an NAD^+^-dependent deacetylase, is decreased in cells resistant to oxaliplatin, which promotes enhanced glycolytic activity. Research suggests that the activation of PARP results in the depletion of NAD^+^ and inhibition of SIRT1; nevertheless, reinstating SIRT1 levels can counteract oxaliplatin resistance and attenuate glycolysis by downregulating glycolytic enzymes such as PKM2 and LDHA, as well as diminishing the extracellular acidification rate [30]. HOTAIR influences the metabolic pathways of colorectal cancer cells by regulating the expression of enzymes tied to glycolysis and glutaminolysis. Modulation of HOTAIR, whether through overexpression or suppression, can alter the levels of enzymes like PKFKB4, PGK1, and LDHA, thus affecting the generation of lactate and glutamate, which are vital metabolites for the survival of tumor cells [27]. Moreover, the upregulation of LGALS4 inhibits aerobic glycolysis in colorectal cancer cells, resulting in decreased glucose dependency and reduced glycolytic activity. This finding underscores LGALS4's inhibitory role in the metabolic processes of these cells [28]. Additionally, the activation of transcription factor 4 (ATF4) can lead to the transcriptional induction of SLC1A5, which enhances both glutaminolysis and glycolysis in colorectal cancer cells. In contrast, the knockdown of SLC1A5 can diminish cell viability, migration, invasion, and glucose metabolism while also lowering the expression of critical glycolytic enzymes such as HK2 and PKM2 [31].

### 3.2. The Impact of Glycolytic Key Enzymes on the Biological Behavior of Colorectal Cancer Cells

Key enzymes that participate in glycolysis are closely linked to the expansion of colorectal cancer cells. FTO is elevated in colon cancer and promotes glycolysis by modulating PKM2, which in turn affects the growth, invasion, and metastasis of colon cancer cells. Research has shown that increased levels of FTO enhance both PKM2 protein expression and the intensity of fluorescence staining for PKM2 in the nucleus, indicating that FTO is vital in the advancement of colorectal cancer through the regulation of PKM2 [32]. In contrast, LGALS4 is found to be downregulated in colorectal cancer, and its increased expression can impede cellular growth, trigger cell cycle arrest, augment apoptosis induced by 5-fluorouracil, inhibit aerobic glycolysis, and diminish cell proliferation. Studies reveal that heightened LGALS4 expression can lead to a reduction in cell proliferation by nearly 50% and double the apoptosis rate, emphasizing its role in suppressing the growth of colorectal cancer cells [28]. Moreover, an examination of genes linked to glycolysis has shown that IER3 and AGRN are significantly overexpressed in colorectal cancer and correlate with lower survival rates among patients. Protein–protein interaction (PPI) analyses indicate that these genes act as central hub genes within the glycolysis pathway, highlighting their importance in the growth of colorectal cancer cells and the progression of the disease [33]. Furthermore, TRIP13 is markedly expressed in colorectal cancer and can trigger glycolysis to enhance cell stemness, thus increasing the resistance of colorectal cancer cells to doxorubicin. Studies in animals have also supported its role in promoting tumor growth [34]. Key enzymes that are essential in glycolysis have a critical function in the invasion and metastasis of colorectal cancer. The long non-coding RNA (lncRNA) FTX elevates the levels of YAP1 by acting as a sponge for miR-215-3p, which in turn enhances aerobic glycolysis, cellular proliferation, migration, and invasion in colorectal cancer cells. This has been confirmed through various experiments conducted both in vitro and in vivo [26]. HOTAIR is involved in the modulation of glycolysis and glutaminolysis within colorectal cancer cells. By affecting the expression of related enzymes, it is vital for preserving the bioenergy and biomass of tumor cells, which can subsequently influence the invasion and metastasis of colorectal cancer [27]. Studies suggest that an elevated glycolytic and lipid metabolic condition is associated with a poor prognosis in patients diagnosed with stage III colorectal cancer. The increased expression of key regulatory genes, including GLUT1, PKM2, FASN, and SCD1, significantly promotes the growth and migration of colorectal cancer cells and enhances resistance to oxaliplatin [35]. In the acidic environment of tumors, ASIC3 triggers epithelial–mesenchymal transition (EMT) through the regulation of essential enzymes involved in de novo lipid synthesis, specifically ACC1 and SCD1, thereby promoting the invasion and metastasis of colorectal cancer cells. Its diminished expression in colorectal cancer is associated with metastasis and staging [36]. Additionally, LINC01977 stimulates aerobic glycolysis via the ERK/c-Myc pathway, boosting the proliferation and metastasis of colorectal cancer cells. It shows a significant increase in colorectal cancer tissues and cell lines, correlating with aggressive clinicopathological traits and a poor prognosis [37].

## 4. The Role of Glycolytic Key Enzymes in the Diagnosis of Colorectal Cancer

Research findings indicate that various crucial enzymes involved in glycolysis have the capacity to function as biomarkers for colorectal cancer. An examination of genetic information from patients with colorectal cancer disclosed that IER3 and AGRN, which play essential roles in the glycolytic pathway, are notably upregulated in colorectal cancer and are linked to reduced survival rates among patients. Analyzing the PPI network revealed these genes as central hubs within the glycolytic framework, and ROC curve analysis validated their ability to distinguish colorectal cancer patients from healthy individuals, highlighting their potential as markers for prognosis, diagnosis, and therapeutic targets in colorectal cancer [33]. Utilizing RNA sequencing data from colorectal cancer patients, a prognostic model that included 14 lncRNAs associated with glycolysis was established, allowing for the stratification of patients into low-risk and high-risk categories. Those identified as low-risk showed extended overall survival. The model demonstrated excellent sensitivity and specificity, with lncRNAs like TNFRSF10A-AS1 and ZKSCAN2-DT effectively indicating patient prognosis. This suggests that lncRNAs linked to glycolysis could be important prognostic and therapeutic biomarkers in colorectal cancer [38]. Moreover, TRIP6 exhibits significant upregulation in colorectal cancer and correlates with various disease stages. Functional enrichment analysis implies its role in focal adhesion and glycolysis, potentially influencing its oncogenic impact by regulating the glycolysis-related gene GPI. Furthermore, its expression correlates with immune cell infiltration and angiogenesis, suggesting its viability as a biomarker for both the diagnosis and treatment of colorectal cancer [39].

## 5. The Application of Key Glycolytic Enzymes in the Treatment of Colorectal Cancer

Therapeutic approaches aimed at crucial enzymes involved in glycolysis present new prospects for treating colorectal cancer. Research has revealed that oxaliplatin-resistant colorectal cancer cells exhibit reduced levels of SIRT1 expression, which results in heightened glycolysis activity. Restoring the expression of SIRT1 may inhibit glycolysis, thus potentially overcoming resistance to oxaliplatin and offering a viable strategy to tackle drug resistance in colorectal cancer [30]. Itraconazole, recognized as an antifungal medication, has been proven to curtail both glycolysis and tumor proliferation in colorectal cancer cells through the CEBPB-ENO1 pathway. Clinical research shows a significant increase in CEBPB expression among colorectal cancer patients, which correlates with unfavorable survival rates, suggesting that itraconazole could represent a new therapeutic avenue for managing colorectal cancer [40]. Furthermore, several natural compounds have demonstrated efficacy in combating colorectal cancer by focusing on the glycolytic pathway. For instance, hesperidin has been shown to lower the levels of essential glycolytic enzymes in colorectal cancer cells, such as hexokinase 2 and glucose transporter 1, thus hindering cell proliferation and migration while encouraging apoptosis. Both in vitro and in vivo studies have established its inhibitory impact on colorectal cancer, highlighting its potential as a therapeutic agent for this condition [41]. Progress has been noteworthy in the development of inhibitors that specifically target essential enzymes involved in glycolysis. The investigation of glucose transporter (GLUT) inhibitors has garnered significant attention due to the rapid glucose uptake and enhanced glycolysis observed in cancer cells. Given that GLUTs are crucial proteins facilitating transmembrane glucose transport, they have emerged as promising therapeutic targets in oncology. An analysis from a medicinal chemistry perspective has examined the advancements, design strategies, and challenges encountered in the formulation of GLUT inhibitors, yielding critical insights for ongoing research and development efforts. However, various challenges remain, particularly in enhancing the selectivity and efficacy of these inhibitors [42]. Additionally, research is progressing on inhibitors that target the mitochondrial pyruvate carrier (MPC). MPC is integral to cellular energy metabolism, and its dysfunction has been linked to multiple metabolic disorders and cancers. Presently, there is a solid understanding of MPC's structure, regulatory framework, and biological roles, with some advances achieved in devising therapeutic drugs aimed at MPC. Nonetheless, the design of selective and potent inhibitors remains challenging, with contemporary virtual screening and computational techniques providing ways to identify new inhibitors [43]. Research on inhibitors targeting key glycolytic enzymes is currently ongoing, with several candidates having entered clinical trials. For example, dichloroacetate, an inhibitor of pyruvate dehydrogenase kinase, has progressed to clinical trials and has exhibited antitumor activity in glioblastoma patients when administered alongside temozolomide and radiotherapy [44]. Similarly, BAY-876, a selective inhibitor of glucose transporter 1, has shown potential in preclinical models, particularly in CRC cells, where it synergizes with mitochondrial complex I inhibitors to inhibit tumor growth [45]. Nevertheless, further validation of its safety and efficacy is essential to confirm its practical application in colorectal cancer treatment.

## 6. The Relationship Between Key Glycolytic Enzymes and the Immune Microenvironment in Colorectal Cancer

The connection between major glycolytic enzymes and the immune microenvironment in colorectal cancer is both intricate and debated. On one side, research suggests that an increase in glycolysis within tumor cells may result in the buildup of lactate in the tumor microenvironment, which suppresses the functionality of T cells and natural killer (NK) cells, thus facilitating immune evasion. For example, metabolic alterations linked to lactate in colorectal cancer influence the performance of immune cells, and higher levels of lactate are correlated with tumor cell spread, the formation of new blood vessels, and resistance to treatment. Modulating key glycolytic enzymes to decrease lactate production may enhance the immune environment [46]. Conversely, the precise mechanisms through which key glycolytic enzymes influence the infiltration, activation, and functions of immune cells remain inadequately understood. For example, PKM2 has the ability to modulate PD-L1 levels in M2 macrophages within the context of tumor immunity, potentially leading to a reduction in both the quantity and activity of CD8^+^ T cells. However, the specific roles of PKM2 across different immune cell types and its interactions with other immune regulatory factors are still not fully elucidated [47]. Furthermore, the gut microbiota plays a crucial role in the onset and progression of colorectal cancer. The complex interplay between the gut microbiota, important glycolytic enzymes, and the immune microenvironment calls for additional comprehensive research. Clarifying how these factors interact will support the advancement of more effective immunotherapy approaches for colorectal cancer. Recent studies have combined crucial glycolytic enzymes with immunotherapy, creating fresh possibilities for treating colorectal cancer. The findings demonstrated that enolase 1 (ENO1), modified by O-GlcNAcylation in colorectal cancer, simultaneously influences aerobic glycolysis and immune evasion. Enhanced glycolytic activity of ENO1 is due to glycosylation at the T19 site, whereas glycosylation at the S249 site contributes to the stabilization of PD-L1 and prevents its interaction with the E3 ligase STUB1. Disabling glycosylation at these two sites can reduce glycolysis and improve T cell-mediated anti-tumor immune responses, respectively, and works synergistically with PD-L1 monoclonal antibody treatment, thereby presenting an innovative approach to colorectal cancer therapy [48]. The combination of chemotherapy and glycolysis-targeted treatment can further boost immunotherapeutic effectiveness in colorectal cancer. For example, self-assembled nano-PROTACs (DdLD NPs) can decrease glycolysis in colorectal cancer, downregulate PD-L1 expression, and concurrently prompt immunogenic cell death through chemotherapy, initiating systemic anti-tumor immunity and significantly averting the advancement of both primary and metastatic colorectal cancer while minimizing notable systemic side effects, thus offering a novel strategy for merging chemotherapy with immunotherapy [49]. Additionally, various studies have suggested that adjusting key glycolytic enzymes can affect the infiltration and effectiveness of immune cells within the tumor microenvironment. For example, inhibiting essential glycolytic enzymes can modify the composition and function of immune cells in the tumor microenvironment, thereby improving the efficacy of immunotherapy. This offers further avenues for comprehensive colorectal cancer treatment.

## 7. Conclusion

This review summarizes the multifaceted roles of key glycolytic enzymes in the initiation, progression, diagnosis, and therapeutic transformation of colorectal cancer. It emphasizes that metabolic reprogramming not only provides energy and biosynthetic precursors for tumor cells but also influences the tumor immune microenvironment and therapeutic responses through metabolites and post-translational modifications. Future research should leverage multi-omics and spatially resolved technologies to elucidate tumor metabolic heterogeneity, focusing on toxicity and metabolic compensation mechanisms to facilitate the translation of mechanistic discoveries into safe and effective clinical interventions. Through these approaches, precise interventions targeting glycolysis are anticipated to become significant supplementary strategies for enhancing CRC prognosis and overcoming therapeutic resistance.

## Data Availability

The data that support the findings of this study are available from the corresponding author upon reasonable request.

## References

[1] Tian S., Chen X., Li J. (2025). Zinc Finger Transcription Factor ZNF24 Inhibits Colorectal Cancer Growth and Metastasis by Suppressing MMP2 Transcription. *Genes & Diseases*.

[2] Yu J., Xiu M., Yu S., Chen Z., Li Y., Gao Y. (2025). The Role of CAF-Derived Vitronectin in Promoting Colorectal Cancer Progression and Immunosuppression. *Advanced Science*.

[3] Gu J., Wang Y., Zhang H., Gu H., Zhu H. (2025). SIGLEC1 Has the Potential to Be an Immune-Related Prognostic Indicator in Colon Adenocarcinoma: a Study Based on Transcriptomic Data and Mendelian Randomization Analysis. *Discover Oncology*.

[4] Karbalaei Hashemiyan M., Manouchehri Ardekani R., Razavizade M., Rafat A., Hemmati-Dinarvand M. (2025). Plasma Expression of Antisense LncRNAs RBM5-AS1, VPS9D1-AS1 and STEAP3-AS1 AS Novel Biomarkers for Colorectal Cancer Diagnosis. *Molecular Biology Reports*.

[5] Rumpold H., Niedersüß-Beke D., Heiler C. (2020). Prediction of Mortality in Metastatic Colorectal Cancer in a Real-Life Population: a Multicenter Explorative Analysis. *BMC Cancer*.

[6] Bonnay F., Veloso A., Steinmann V. (2020). Oxidative Metabolism Drives Immortalization of Neural Stem Cells During Tumorigenesis. *Cell*.

[7] Gaude E., Frezza C. (2016). Tissue-Specific and Convergent Metabolic Transformation of Cancer Correlates With Metastatic Potential and Patient Survival. *Nature Communications*.

[8] Chen Y., Zhu H., Luo Y. (2025). ALDOC Promotes Neuroblastoma Progression and Modulates Sensitivity to Chemotherapy Drugs by Enhancing Aerobic Glycolysis. *Frontiers in Immunology*.

[9] Lee R., Won K. J., Kim J. H., Lee B. H., Hwang S. H., Nah S. Y. (2024). Gintonin Stimulates Glucose Uptake in Myocytes: Involvement of Calcium and Extracellular Signal-Regulated Kinase Signaling. *Biomolecules*.

[10] Zeng Y., Tao Y., Du G. (2025). Advances in the Mechanisms of HIF-1*α*-Enhanced Tumor Glycolysis and Its Relation to Dedifferentiation. *Progress in Biophysics and Molecular Biology*.

[11] Liang P., Li Z., Chen Z. (2025). Metabolic Reprogramming of Glycolysis, Lipids, and Amino Acids in Tumors: Impact on CD8+ T Cell Function and Targeted Therapeutic Strategies. *The FASEB Journal*.

[12] Xu L., Shen Y., Zhang C., Shi T., Sheng X. (2025). Exploring the Link Between Noncoding RNAs and Glycolysis in Colorectal Cancer. *Journal of Cellular and Molecular Medicine*.

[13] Chen J., Chao D., Tran U. P., Billingsley K. L. (2024). Design, Synthesis, and Assessment of Tricarboxylic Acid Cycle Probes. *Synthesis*.

[14] Jiang H., Ye J. (2025). The Warburg Effect: The Hacked Mitochondrial-Nuclear Communication in Cancer. *Seminars in Cancer Biology*.

[15] Papaneophytou C. (2024). The Warburg Effect: Is It Always an Enemy?. *Frontiers in Bioscience-Landmark*.

[16] Warburg O., Wind F., Negelein E. (1927). The Metabolism of Tumors in the Body. *The Journal of General Physiology*.

[17] Hanahan D., Weinberg R. A. (2011). Hallmarks of Cancer: The Next Generation. *Cell*.

[18] Lunt S. Y., Vander Heiden M. G. (2011). Aerobic Glycolysis: Meeting the Metabolic Requirements of Cell Proliferation. *Annual Review of Cell and Developmental Biology*.

[19] Cordani M., Michetti F., Zarrabi A. (2024). The Role of Glycolysis in Tumorigenesis: From Biological Aspects to Therapeutic Opportunities. *Neoplasia*.

[20] Vander Heiden M. G., Cantley L. C., Thompson C. B. (2009). Understanding the Warburg Effect: The Metabolic Requirements of Cell Proliferation. *Science*.

[21] Zhou M., Qin Z., Zhu X. (2025). Pyruvate Dehydrogenase Kinases: Key Regulators of Cellular Metabolism and Therapeutic Targets for Metabolic Diseases. *Journal of Physiology and Biochemistry*.

[22] Liu S., Shen G., Zhou X. (2024). Hsp90 Promotes Gastric Cancer Cell Metastasis and Stemness by Regulating the Regional Distribution of Glycolysis-Related Metabolic Enzymes in the Cytoplasm. *Advanced Science*.

[23] Lara-Núñez A., Guerrero-Molina E. D., Vargas-Cortez T., Vázquez-Ramos J. M. (2024). Interplay of CDKs and Cyclins With Glycolytic Regulatory Enzymes PFK and PK. *Journal of Plant Physiology*.

[24] Malla A., Gupta S., Sur R. (2024). Glycolytic Enzymes in Non-Glycolytic Web: Functional Analysis of the Key Players. *Cell Biochemistry and Biophysics*.

[25] Xu W., Weng J., Xu M. (2023). Functions of Key Enzymes of Glycolytic Metabolism in Tumor Microenvironment. *Cellular Reprogramming*.

[26] Yang J. L., Ma J. J., Qu T. Y. (2025). Glycolysis-Related lncRNA FTX Upregulates YAP1 to Facilitate Colorectal Cancer Progression via Sponging miR-215-3p. *Scientific Reports*.

[27] Flores-García L. C., García-Castillo V., Pérez-Toledo E. (2025). HOTAIR Participation in Glycolysis and Glutaminolysis Through Lactate and Glutamate Production in Colorectal Cancer. *Cells*.

[28] Li S., Yang K., Ye J. (2025). LGALS4 Inhibits Glycolysis and Promotes Apoptosis of Colorectal Cancer Cells Via *β*-Catenin Signaling. *Oncology Letters*.

[29] Chen J., Duan S., Wang Y. (2024). MYG1 Drives Glycolysis and Colorectal Cancer Development Through Nuclear-Mitochondrial Collaboration. *Nature Communications*.

[30] Niu Y. R., Xiang M. D., Yang W. W., Fang Y. T., Qian H. L., Sun Y. K. (2025). NAD+/SIRT1 Pathway Regulates Glycolysis to Promote Oxaliplatin Resistance in Colorectal Cancer. *World Journal of Gastroenterology*.

[31] Zhou Z., Ye S., Chen J. (2025). ATF4 Promotes Glutaminolysis and Glycolysis in Colorectal Cancer by Transcriptionally Inducing SLC1A5. *Acta Biochimica et Biophysica Sinica*.

[32] Zhang K., Zhang F., Wang J. (2025). FTO Effects the Proliferation, Invasion, and Glycolytic Metabolism of Colon Cancer by Regulating PKM2. *Journal of Cancer Research and Clinical Oncology*.

[33] Dalali S., Kaviani F., Mahdevar M., Oroujalian A., Peymani M. (2025). Analysing Glycolysis-Related Genes Reveals the Prognostic and Diagnostic Relevance of IER3 and AGRN in Colorectal Cancer. *Genes Genomics*.

[34] Liu G., Wang H., Ran R., Wang Y., Li Y. (2024). TRIP13 Activates Glycolysis to Promote Cell Stemness and Strengthen Doxorubicin Resistance of Colorectal Cancer Cells. *Current Medicinal Chemistry*.

[35] Li M., Yue M., Chen Y., Zhao G. (2025). High Glycolysis and Lipid Metabolism Status Predicts Poor Prognosis in Colorectal Cancer Patients. *Current Molecular Medicine*.

[36] Wan X., Li F., Li Z., Zhou L. (2024). ASIC3-Activated Key Enzymes of De Novo Lipid Synthesis Supports Lactate-Driven EMT and the Metastasis of Colorectal Cancer Cells. *Cell Communication and Signaling: CCS*.

[37] Wu J., Chen Q., Wang Y. (2024). LINC01977 Promotes Colorectal Cancer Growth and Metastasis by Enhancing Aerobic Glycolysis via the ERK/c-Myc Axis. *Journal of Gastrointestinal Oncology*.

[38] Zhu G., Kang Y., Luo M., Ju L., Sun Y., Chen L. (2025). Identification of a Glycolysis-Associated lncRNA Signature to Predict Survival of Patients With Colorectal Cancer. *International Journal of Clinical and Experimental Pathology*.

[39] Liu X. S., Chen Y. X., Wan H. B. (2024). TRIP6 A Potential Diagnostic Marker for Colorectal Cancer With Glycolysis and Immune Infiltration Association. *Scientific Reports*.

[40] Zhang Y., Li L., Chu F. (2024). Itraconazole Inhibits Tumor Growth via CEBPB-Mediated Glycolysis in Colorectal Cancer. *Cancer Science*.

[41] Sun K. X., Tan W. S., Wang H. Y. (2025). Hesperidin Suppressed Colorectal Cancer Through Inhibition of Glycolysis. *Chinese Journal of Integrative Medicine*.

[42] Wang Y., Sun Z., Zhao Z., Pang J., Chen J. (2025). Recent Progress in the Development of Glucose Transporter (GLUT) Inhibitors. *Journal of Medicinal Chemistry*.

[43] Politte H., Maram L., Elgendy B. (2025). Advances in the Development of Mitochondrial Pyruvate Carrier Inhibitors for Therapeutic Applications. *Biomolecules*.

[44] Xintaropoulou C., Ward C., Wise A., Marston H., Turnbull A., Langdon S. P. (2015). A Comparative Analysis of Inhibitors of the Glycolysis Pathway in Breast and Ovarian Cancer Cell Line Models. *Oncotarget*.

[45] Guo L., Zhang W., Xie Y. (2022). Diaminobutoxy-Substituted Isoflavonoid (DBI-1) Enhances the Therapeutic Efficacy of GLUT1 Inhibitor BAY-876 by Modulating Metabolic Pathways in Colon Cancer Cells. *Molecular Cancer Therapeutics*.

[46] Sun Q., Wu J., Zhu G. (2023). Lactate-Related Metabolic Reprogramming and Immune Regulation in Colorectal Cancer. *Frontiers in Endocrinology*.

[47] Chen M., Liu H., Li Z., Ming A. L., Chen H. (2021). Mechanism of PKM2 Affecting Cancer Immunity and Metabolism in Tumor Microenvironment. *Journal of Cancer*.

[48] Zhu Q., Li J., Sun H. (2024). O-GlcNAcylation of Enolase 1 Serves as a Dual Regulator of Aerobic Glycolysis and Immune Evasion in Colorectal Cancer. *Proceedings of the National Academy of Sciences of the United States of America*.

[49] Zhao L. P., Zheng R. R., Rao X. N. (2024). Chemotherapy-Enabled Colorectal Cancer Immunotherapy of Self-Delivery Nano-PROTACs by Inhibiting Tumor Glycolysis and Avoiding Adaptive Immune Resistance. *Advanced Science*.

